# Dendritic Cell Based Tumor Vaccination in Prostate and Renal Cell Cancer: A Systematic Review and Meta-Analysis

**DOI:** 10.1371/journal.pone.0018801

**Published:** 2011-04-20

**Authors:** Andreas Draube, Nela Klein-González, Stefanie Mattheus, Corinne Brillant, Martin Hellmich, Andreas Engert, Michael von Bergwelt-Baildon

**Affiliations:** 1 Laboratory for Tumor and Transplantation Immunology, Department I of Internal Medicine, University Hospital of Cologne, Cologne, Germany; 2 Cochrane Hematological Malignancies Group, Department I of Internal Medicine, University Hospital of Cologne, Cologne, Germany; 3 Institute of Medical Statistics, Informatics and Epidemiology (IMSIE), University of Cologne, Cologne, Germany; 4 Center for Molecular Medicine (CMMC), University of Cologne, Cologne, Germany; National Cancer Institute, United States of America

## Abstract

**Background:**

More than 200 clinical trials have been performed using dendritic cells (DC) as cellular adjuvants in cancer. Yet the key question whether there is a link between immune and clinical response remains unanswered. Prostate and renal cell cancer (RCC) have been extensively studied for DC-based immunotherapeutic interventions and were therefore chosen to address the above question by means of a systematic review and meta-analysis.

**Methodology/Principal Findings:**

Data was obtained after a systematic literature search from clinical trials that enrolled at least 6 patients. Individual patient data meta-analysis was performed by means of conditional logistic regression grouped by study. Twenty nine trials involving a total of 906 patients were identified in prostate cancer (17) and RCC (12). Objective response rates were 7.7% in prostate cancer and 12.7% in RCC. The combined percentages of objective responses and stable diseases (SD) amounted to a clinical benefit rate (CBR) of 54% in prostate cancer and 48% in RCC. Meta-analysis of individual patient data (n = 403) revealed the cellular immune response to have a significant influence on CBR, both in prostate cancer (OR 10.6, 95% CI 2.5–44.1) and in RCC (OR 8.4, 95% CI 1.3–53.0). Furthermore, DC dose was found to have a significant influence on CBR in both entities. Finally, for the larger cohort of prostate cancer patients, an influence of DC maturity and DC subtype (density enriched versus monocyte derived DC) as well as access to draining lymph nodes on clinical outcome could be demonstrated.

**Conclusions/Significance:**

As a ‘proof of principle’ a statistically significant effect of DC-mediated cellular immune response and of DC dose on CBR could be demonstrated. Further findings concerning vaccine composition, quality control, and the effect of DC maturation status are relevant for the immunological development of DC-based vaccines.

## Introduction

For more than a century it has been hypothesized that the immune system can be redirected to target malignant cells and thus cure cancer [1], [2]. This concept is based on the notion of cancer immunosurveillance [3]. In accordance to this concept it was shown that in patients with colon cancer the density of infiltrating, antigen-experienced T cells correlated better with clinical outcome than classical histopathological staging [4]. Furthermore, it could be demonstrated that people with a high degree of natural cytotoxicity had a lower risk to develop cancer [5].

Dendritic cells (DC) [6] play a crucial role in the induction of antigen-specific T-cell responses and are therefore the most frequently used cellular adjuvant in clinical trials. However, the heterogeneity of vaccination algorithms, non-standardized cellular products and the lack of established criteria for clinical and immune responses has made it impossible to draw valid conclusions from single clinical trials [7]–[10]. The key questions about whether induction of immunity is linked to clinical response and whether a dose-response relationship exists remain unanswered. Prostate and renal cell cancer (RCC) are regularly infiltrated by antigen-specific immune cells and are considered susceptible to immunotherapy [11]–[13]. DC vaccination has been extensively studied in these cancers [14]–[18]. Additionally, the clinical response criteria used for these tumor entities are complementary: Conventional RECIST/WHO criteria in RCC and combination of radiographic criteria with biochemical markers in prostate cancer. For these reasons, they were identified as an ideal model for a systematic review of DC-based tumor vaccines. The aim of this study was to analyze the studies identified in the literature search concerning vaccination strategies, quality control, and induction of immune and clinical response. Furthermore, using individual-patient data the hypothesis was tested whether induction of immunity indeed leads to disease control. Additionally, immunologic properties with potential influence on vaccination success (e.g. maturation status, access to draining lymph nodes) were analyzed.

## Methods

### Literature search, inclusion and exclusion criteria

A highly sensitive search string for Medline-Ovid was developed in which keywords related to ‘cancer’, ‘dendritic cells’, and ‘clinical trial’ were combined (Text S1). The Medline database search covered the period from January 1987 to September 2008, and was recently up-dated covering the period until December 2010. The search was performed in accordance to the PRISMA statement [19] which is focused on meta-analysis of randomized trials, but can be used as a general basis for systematic reviews.

Clinical trials using DC in prostate and RCC, that enrolled at least 6 patients, published between January 2000 and December 2010, and in English language were included. Studies in which different tumor entities were treated, follow-up studies, and trials using allogeneic DC were excluded.

### Study selection and data extraction

Two reviewers (NKG and SM) independently screened the articles identified in the literature search. Disagreements between the reviewers (about 15% of the cases) were resolved by a consensus involving two other reviewers (AD, MBB). Data were collected both at trial and individual patient level in an Access database for: DC subtype, phenotype and purity, antigen delivery, route of vaccination, dose (number of DC per vaccination * number of vaccinations), adjuvants given, toxicity, immune monitoring, and clinical response. Additionally, the following individual patient information was recorded: age, gender, staging, pretreatment, and concomitant therapy. Although information given on phenotype regarding the maturation status of DC was documented, it was the maturation status indicated by the authors that was assumed for the meta-analysis. A cellular or humoral immune response was considered positive when at least one method led to a positive result for the specific antigen, if had not already been detected before vaccination. For clinical outcome, complete response (CR), partial response (PR), mixed response (MR), stable disease (SD), and progressive disease (PD) were documented. The term clinical benefit rate (CBR) was used representing the rate of patients who were either in CR, PR, MR, or SD [20].

### Statistical methods

Descriptive analyses were performed both at study data level and at individual patient data level. Prostate and RCC trials were analyzed separately. Available individual patient data was used for meta-analysis. Variables were dichotomized: 1) DC subtype: mature monocyte-derived DC (mat-moDC) *vs.* immature monocyte-derived DC (imm-moDC); 2) route: intravenous (i.v.) *vs.* intradermal (i.d.)/intranodal (i.n.)/intralymphatic (i.l)/subcutaneous (s.c.); 3) total DC dose for prostate cancer: ≥86.4×10^6^ (median) *vs.* <86.4×10^6^; 4) total DC dose for RCC: ≥38.7×10^6^ (median) *vs.* <38.7×10^6^; 5) age ≥65 *vs.* <65; 6) clinical response: PD *vs.* CR, PR, MR, SD; 7) cellular and 8) humoral immune response: positive *vs.* negative.

Influences on clinical outcome of cellular immune response, humoral immune response, total DC dose, and age were assessed by means of conditional logistic regression grouped by study, thus adjusting varying vaccination protocols or response criteria. Odds ratios (OR) with 95% confidence intervals were calculated. Additionally, the influence of the continuous variable “logarithm of total DC dose (logdose)” on clinical outcome was assessed. Forest plots were supplemented with the overall Mantel-Haenszel estimate (fixed effect).

Data on variables constant within trials, i.e. DC subtype and route of vaccination, were pooled and analyzed by Pearson's chi-square test (unstratified). P-values smaller than 0.05 were considered to indicate statistical significance though no correction for multiple testing was applied. Statistical analyses were performed in IBM SPSS Statistics 19.0 (IBM SPSS Statistics Inc., Chicago, IL), Stata 11.1 (StataCorp, College Station, TX) or Comprehensive Meta Analysis 2.2 (Biostat, Englewood, NJ).

## Results

### Identification of studies

**Figure 1:**
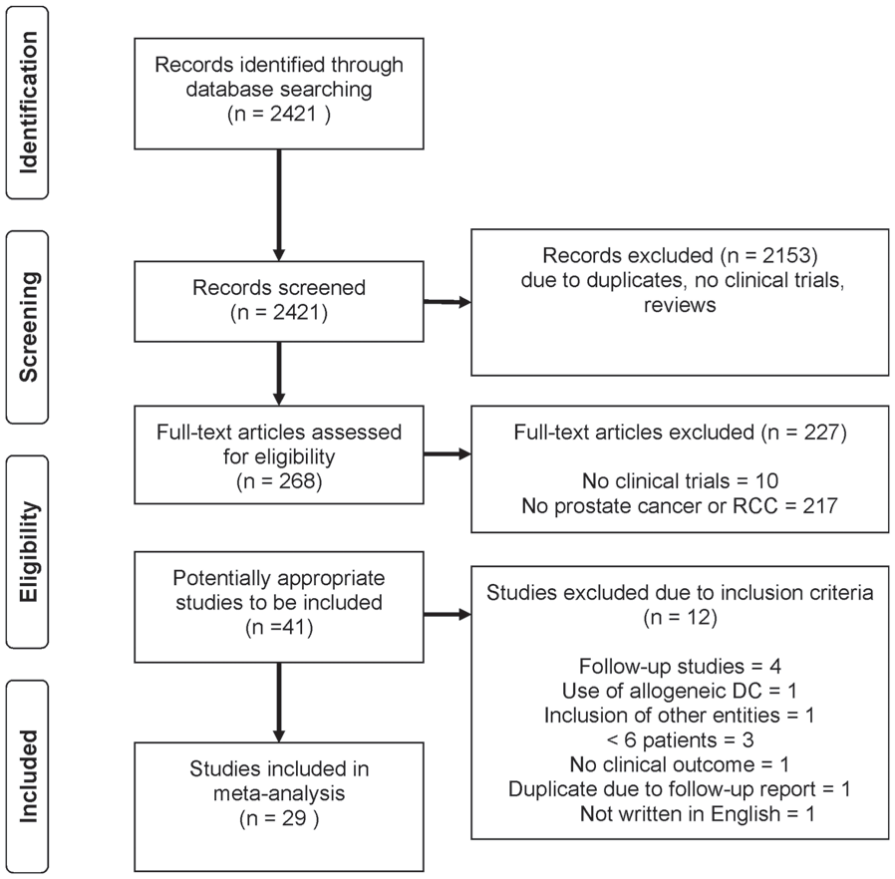
Flow diagram showing record identification, record screening, full text article eligibility and study inclusion process.

Of 268 articles identified as reports about clinical trials, 41 were conducted in patients with *prostate cancer* or *RCC*. Among these, 12 were excluded for the following reasons: follow-up studies (4) [21]–[24]; use of allogeneic DC (1) [25]; inclusion of two tumor entities (1) [26]; <6 patients (3) [27]–[29]; not written in English (1) [30]; lack of information about clinical outcome (1) [31]; and follow-up reporting of the same study (1) [32], [33]. One study included both prostate and *RCC* patients; however, only *prostate cancer* patients were included because the number of patients with *RCC* was only five [34]. Figure 1 displays the search process.

Twenty nine studies in prostate (17) and *RCC* (12) met all inclusion criteria and were subject of this meta-analysis [33]–[61]. In total, the studies included 906 patients (*prostate cancer* 720, *RCC* 186). Apart from three randomized phase III studies in *prostate cancer* [33], [40], [42], all trials were phase I/II clinical studies. The histological *RCC* subtype was specified in 10 of 12 studies and was either clear cell in all patients [51], [52], [54], [59], [61] or clear cell in the vast majority of patients with few exceptions (in all studies <15%: papillary, clear cell/sarcomatoid or chromophilic) [50], [53], [55], [57], [58]. The median patient number within the phase I/II studies was 14 for *prostate cancer* (range 6–31) and 12 for *RCC* (range 8–35) (Table 1).

**Table 1:** Clinical trials with DC based tumor vaccination in prostate cancer and RCC.

Prostate cancer									
Reference	Patients	DC	Antigen (+ helper antigen)	Antigen processing	Adjuvant	Route	Humoral immune response*	Cellular immune response*	Clinical response+
Barrou, 2004 [35]	24	imm-moDC	rPSA	protein		i.v.+s.c.+i.d.	0/24	11/24	11/24 SD
Burch, 2000 [36]	13	density enriched	PA2024§	protein	PA2024 s.c.	i.v.	11/11	9/9	3/13 PR, 9/13 SD
Fong, 2001 [37]	21	density enriched	muPAP	protein		i.v. or i.d. or i.l.	10/21	21/21	6/21 SD
Fuessel, 2006 [38]	8	mat-moDC	PSA3, PSMA1, prostein, survivin, Trp-p8	peptide		i.v. + i.d.	n.d.	4/8	1/8 PR, 3/8 SD
Heiser, 2002 [39]	13	imm-moDC	PSA	mRNA		i.v. + i.d.	0/13	10/10	n.d.
Higano, 2009 [40] ‡	65 ‡	density enriched	PA2024§	protein		i.v.	n.d.	n.d.	n.d.
Hildenbrand, 2007 [41]	12	mat-moDC	PSA-1, PSA-2, PSA-3	peptide	IFN-gamma	s.c.	n.d.	10/12	1/12 PR, 1/12 MR, 4/12 SD
Kantoff, 2010 [42]	341	density enriched	PA2024§	protein		i.v.	100/151	46/63	n.d.
Mu, 2005 [43]	19	mat-moDC	cell lines DU145, LNCaP, PC-3	mRNA		i.d. or i.n.	n.d.	13/19	11/19 SD
Murphy, 2000 [44]	28	imm-moDC	PSM-P1 + PSM-P2 (+ KLH)	peptide		i.v.	n.d.	n.d.	1/28 CR, 4/28 PR, 1/28 SD
Pandha, 2004 [34]	11	imm-moDC	cell lines LNCaP, DU145	cell lysate	KLH i.d.	i.d. or i.n.	n.d.	11/11	4/11 SD
Perambakam, 2006 [45]	14	imm-moDC	PSA (+ Flu-M1)	peptide		i.v.	n.d.	5/14	n.d.
Small, 2000 [46]	31	density enriched	PA2024 (+ KLH in 5 patients)	protein		i.v.	16/31	31/31	3/31 PR, 28/31 SD**
Small, 2006 [33]	82	density enriched	PA2024§	protein		i.v.	n.d.	n.d.	n.d.
Su, 2005 [47]	20	mat-moDC	hTERT or LAMPhTERT	mRNA		i.d.	n.d.	17/18	n.d.
Thomas-Kaskel, 2006 [48]	12	mat-moDC	PSCA, PSA (+ HIV-gag)	peptide		s.c.	n.d.	5/12	6/12 SD
Waeckerle-Men, 2006 [49]	6	mat-moDC	PSCA, PAP, PSMA, PSA	peptide	DC + FluM	i.d.	n.d.	3/4	0/3

RCC									
Reference	Patients	DC	Antigen (+ helper antigen)	Antigen processing	Adjuvant	Route	Humoral immune response*	Cellular immune response*	Clinical response+
Berntsen, 2008 [50]	27	mat-moDC	survivin, telomerase (+ PADRE) or cell lines A-498, Caki-1, Caki-2 (+ KLH)	peptide or tumor lysate	IL2	i.n. or i.d.	n.d.	6/19	13/27 SD
Bleumer, 2007 [51]	8	mat-moDC	CA9p254 + CA9p249 (+ KLH)	peptide		i.d.	0/6	6/6	0/6
Danull, 2005 [52]	10	mat-moDC	auologous tumor cells	total RNA	DAB_389_IL-2 in 6 patients (T_reg_ depletion)	i.d.	n.d.	9/10	n.d.
Gitlitz, 2003 [53]	12	imm-moDC	autologous tumor cells	tumor lysate		i.d.	n.d.	0/12	1/12 PR, 3/12 SD
Höltl, 2002 [54]	35	mat-moDC	autologous tumor cells or cell line A-498 (+ KLH)	tumor lysate		i.v. or i.d.	n.d.	5/6	2/35 CR, 1/35 PR, 7/35 SD
Kim, 2007 [55]	9	mat-moDC	autologous tumor cells (+ KLH)	tumor lysate		s.c.	n.d.	9/9	1/9 PR, 5/9 SD
Märten, 2002 [56]	15	mat-moDC	autologous tumor cells # (+ KLH in 2 patients)	tumor lysate		s.c. or i.n.	n.d.	2/9	1/15 PR, 7/15 SD (2 patients with SD received only KLH pulsed DC)
Oosterwijk-Wakka, 2002 [57]	12	imm-moDC	autologous tumor cells (+ KLH in 6 patients)	tumor lysate	IL2	i.d.	0/12	0/12	8/12 SD
Schwaab, 2009 [58]	18	mat-moDC	autologous tumor cells	tumor lysate	IL2, IFN-α 2a	i.n.	increase of anti-RCC IgM	increase of RCC specific CD8+IFNg+Tcells	3/18 CR, 6/18 PR, 6/18 SD
Su, 2003 [59]	10	imm-moDC	autologous tumor cells	total RNA		i.v. + i.d.	n.d.	6/7	1/2 SD
Wei, 2007 [60]	10	mat-moDC	autologous tumor cells	cell fusion	IL2	s.c.	n.d.	9/9	1/10 PR, 3/10 SD
Wierecky, 2006 [61]	20	mat-moDC	MUC-1 (+ PADRE)	peptide	IL2	s.c.	n.d.	11/18	1/20 CR, 2/20 PR, 2/20 MR, 5/20SD

Abbreviations: mat-moDC, mature monocyte derived dendritic cell; imm-moDC, immature monocyte derived dendritic cell; id, intradermal; in, intranodal; il, intralymphatic; sc, subcutaneous; iv, intraveneous; CR, complete response; PR, partial response; MR, mixed response; SD, stable disease; PD, progressive disease; n.d., not done.

*tumor antigen specific immune responses; details on all conducted tests are given in Table S4.

+ for prostate cancer trials in which PSA courses were used for response criteria they are displayed if they were combined with radiological criteria; clinical responses are indicated in relation to evaluable patients – additional clinical response information and trials response criteria are given in Table S1.

§ PA2024  =  huPAP/GM-CSF fusion protein.

# 2 patients received unpulsed and another 2 patients only KLH pulsed DC.

**at least ≥8 weeks from registration.

‡ only results of the trial D9902A displayed; the publication also reported on an integrated analysis including the results of the trial D9901 [33].

### Patient characteristics

In *prostate cancer*, 662 patients had metastatic disease, 51 biochemical relapse, and 7 local recurrence. All *RCC* patients had metastatic disease (Table S1).

87% (458/526 patients with data available) of *prostate cancer* patients had prior surgery or radiotherapy, 17% (109/646) prior chemotherapy, and 96% (632/660) prior hormone therapy or castration. In one *prostate cancer* trial all patients received IFN-γ simultaneously [41].

92% (142/154 patients with data available) of *RCC* patients had prior surgery or radiotherapy, 17% (24/143) prior chemotherapy and 36% (54/151) prior immunotherapy. In 36% (67/182) of *RCC* patients systemic IL-2 [50], [57], [60] or IL2/IFN-α [58] was simultaneously applied to the DC vaccine. In one study, IL2 was given immediately after the last vaccination [61]. In two studies, 3 and 6 patients, respectively, received IL-2, IL-2/IFN-γ or IL2/IFN-γ/5-FU after the end of the vaccination period [56], [59].

### Vaccines

#### Prostate Cancer

Imm-moDC were used in 5 trials (90 patients) [34], [35], [39], [44], [45], whereas mat-moDC were used in 6 trials (77 patients) [38], [41], [43], [47]–[49]. In 3 phase I/II studies (65 patients) [36], [37], [46] and in the 3 phase III studies (488 patients) [33], [40], [42] density-enriched DC were used. Numerous strategies for antigen delivery were employed (Table 1): Peptide pulsing was preferred in 6 studies [38], [41], [44], [45], [48], [49]; loading with whole protein in 7 studies [33], [35]–[37], [40], [42], [46]; loading with tumor lysates in one study [34]; and RNA transfection or coincubation in 3 studies [39], [43], [47]. In 11 studies (87% of all *prostate cancer* patients), vaccination was intravenously applied or an intravenous and a non-intravenous route (i.d., s.c., i.l., i.n.) were combined. In 6 studies (13% of all patients) a non-intravenous route was preferred. Details are compiled in Table 1.

Cell numbers applied per vaccination and numbers of vaccination are given in Table S2.

#### RCC

Imm-moDC were used in 3 studies (34 patients) [53], [57], [59], whereas mat-moDC in 9 studies (152 patients) [50]–[52], [54]–[56], [58], [60], [61]. Peptide pulsing was chosen in two studies [51], [61]; loading with tumor lysates in 6 studies [53]–[58]; RNA coincubation in two studies [52], [59]; and cell fusion with autologous tumor cells in another study [60]. In one study DC were either loaded with peptides or tumor lysate [50]. In 10 studies a non-intravenous route was chosen (84% of all *RCC* patients), whereas in two studies (16% of patients) an intravenous or a combined vaccination was preferred (Table 1).

#### Comparison of vaccines between prostate cancer and RCC trials

In *prostate cancer* trials an intravenous route or a combination of different routes was the strategy most frequently preferred, whereas a non-intravenous route was chosen in the majority of *RCC* trials. In regard to the total patient number most *prostate cancer* patients received density-enriched DC, which was due to the large randomized trials, whereas most *RCC* patients received mat-moDC. In *prostate cancer* trials, pulsing with defined peptides or proteins was the preferred antigen delivery strategy, whereas in *RCC* it was pulsing with tumor cell lysates.

In regard to individual patient data available for the meta-analysis compared to the study data level described above, it is important to mention that for *prostate cancer* the percentages of patients receiving mat-moDC, imm-moDC, and density-enriched DC were more evenly distributed with 33%, 39%, and 28%, respectively. The ratio of intravenous - non-intravenous route was also more evenly distributed: 52% *vs.* 48% (compare to Table 1 and Table S5). In contrast, for *RCC* individual-patient data available, the uneven distribution regarding the route of vaccination and the DC maturation status was the same compared to the study data level described above.

### Quality controls

To determine quality control of the DC-based vaccines, criteria proposed by Figdor and colleagues were followed concerning phenotype and purity [62]. The authors suggested six surface antigens for mat-moDC to be documented (CD83, CD80, CD86, MHC-I, MHC-II, CCR7) and 7 for imm-moDC (CD14, CD83, CD80, CD86, MHC-I, MHC-II, CCR5). No trial using moDC reported all antigens proposed. Nevertheless, in *prostate cancer* two studies reported 5 of 6 and 6 of 7; in *RCC* four studies reported 5 of 6 and 6 of 7 proposed antigens for mat-moDC and imm-moDC, respectively [39], [49]–[51], [55], [59]. On the other hand, two *prostate cancer* studies and three *RCC* studies failed to report the phenotype [34], [44], [53], [56], [60]. The majority of the studies provided information for the following surrogate markers: CD14 (*prostate cancer* 8/17 studies, *RCC* 7/12 studies), HLA-DR (*prostate cancer* 11/17, *RCC* 7/12), CD86 (*prostate cancer* 11/17, *RCC* 8/12), and CD83 (*prostate cancer* 9/17, *RCC* 9/12).

Information about purity of DC vaccine was available only in 10 studies, (*prostate cancer*: 8/17, *RCC*: 2/12). Purity above 80% proposed by Figdor and co-workers was indicated only in four trials (*prostate cancer*: 3/17 and *RCC*: 1/12) [35], [41], [49], [55].

Comparing *RCC* with *prostate cancer* revealed that information on DC phenotype was slightly more detailed in *RCC* trials, whereas the information on DC purity was better in *prostate cancer* trials.

### Treatment related toxicity

DC vaccination was safe in both entities: only mild side effects were described. Most adverse effects were local reactions at the injection site, fever and flu-like symptoms. Less common were myalgias, fatigue, bone or articular pains. Few toxicities were detected above grade 2 or were linked to additional cytokine therapy (e.g. [58]). A detailed overview is provided in Table S3.

### Immune response assessment

The cellular immune response was determined in all studies except for two [40], [44]. At least one of the functional tests proposed by Figdor – ELISPOT, cytotoxicity, or cytokine production after antigen-specific stimulation [62] – was performed in the majority of the studies (*prostate cancer* 10/17; *RCC* trials 10/12). Humoral immune response using antigen-specific ELISA was reported in 6 of 17 *prostate cancer* and in 2 of 12 *RCC* trials.

**Figure 2:**
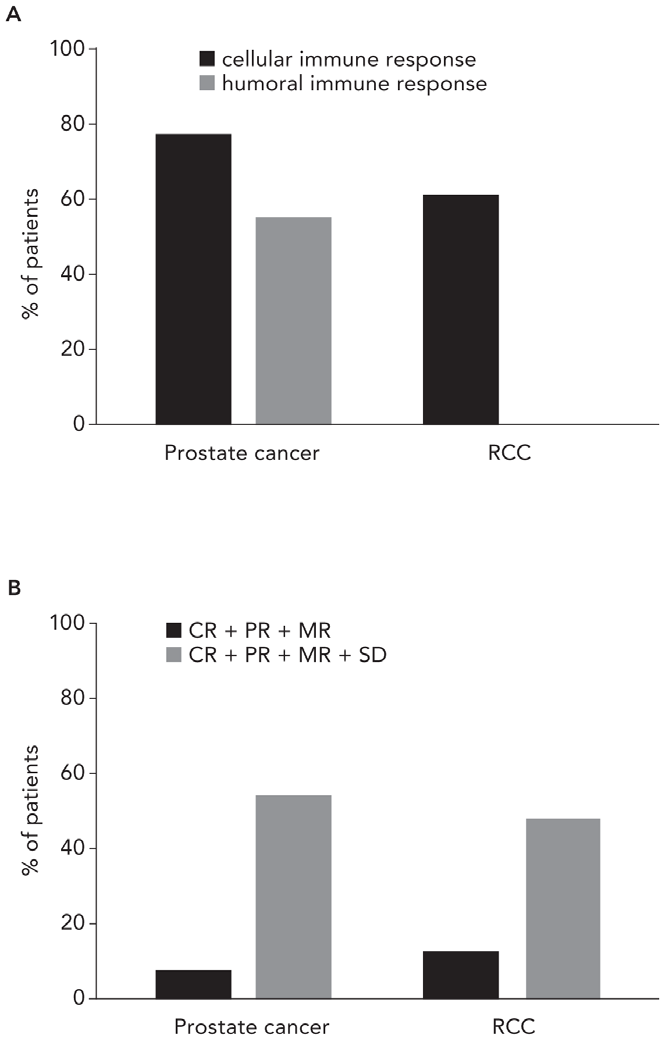
Immune and clinical responses in prostate and renal cell cancer (RCC) patients. (A) Induction of tumor antigen specific cellular or humoral immune responses. An immune response was considered positive when at least one of the conducted assays was positive. (B) Analysis of objective response (black columns) and clinical benefit rate (CBR; grey columns). CR, complete response; PR, partial response; MR, mixed response; SD, stable disease.

Analysis of study data level (Table 1) revealed that DC vaccination led to an antigen-specific cellular immune response in 77% of patients with *prostate cancer* (196/256 patients tested) and in 61% of patients with *RCC* (63/104 patients tested) (Fig. 2A). A specific humoral immune response was detected in 55% of *prostate cancer* patients (137/251 patients tested), whereas no humoral response was observed in the few *RCC* patients tested (0/18 patients tested). Complete information about immune assessment is summarized in Table S4.

### Clinical response

Lack of radiologically measurable disease is a frequent problem in *prostate cancer* trials. The Prostate-Specific Antigen Working Group of the NCI has therefore incorporated PSA levels into remission criteria for metastatic disease [63]. Most studies combined both PSA levels and radiological response [36]–[38], [41], [43], [44], [48]. In 12 of 17 trials ‘progressive disease’ as prerequisite before study entry was explicitly required, whereas in the other 5 trials the study entry was not limited, it was allowed for all patients with metastatic or hormone refractory disease (Table S1). Six trials could not be assessed for clinical response since: 1) clinical response was not reported [45]; 2) only reports on PSA doubling times [39]; 3) median time to progression (TTP) [33], [40], [42]; or 4) median PSA courses of vaccination groups [47] were documented.

In *RCC* trials response criteria were in accordance to RECIST or WHO criteria; however, these were explicitly mentioned only in 5 trials [16], [55], [56], [58], [61]. Eight studies only included patients with documented progressive or new metastatic disease before nephrectomy, in one study 85% of the patients had progressive disease, and in three studies enrollment was allowed to all patients with metastatic disease. In one trial clinical response was not reported [52]. Table S1 contains information about the trials clinical response evaluation.

Overall, the clinical response was documented in 181 *prostate cancer* patients. 7.7% of the patients had an objective response (CR+PR+MR). Additionally, 83 SD were observed representing a clinical benefit rate (CBR) of 54% (97/181) (Fig. 2B). On the other hand, in a total of 166 *RCC* patients investigated, objective response rate was 12.7%. SD was found in 58 patients, resulting in a CBR of 48% (79/166) (Fig. 2B; Table S1).

### Meta-analysis

Individual data was available from 231 *prostate cancer* and 172 *RCC* patients (Table S5). Different variables were tested for their influence on CBR in which stable diseases (SD) were included. Separated stratified analyses for the influence only on CR+PR were not possible due to their low number. Influence of the following variables on CBR was assessed by conditional logistic regression grouped by study: 1) cellular immune response, 2) total DC dose, 3) humoral immune response, 4) age, and 5) gender of *RCC* patients (Table 2). Additionally, the continuous variable DC logdose was assessed. DC subtype and route of vaccination were constant within most of the trials and therefore tested by means of unstratified chi-square tests (Table 3).

**Table 2:** Meta-analysis of influence on clinical benefit rate - conditional logistic regression grouped by study.

Prostate cancer				
Variable	Number of patients^$^	OR	95% CI	p-value(2-sided)
Cellular immune response	83	10.567	2.533–44.082	0.001*
Humoral immune response	21	0.760	0.128–4.522	0.763
Median dose (≥86.4 *vs.* <86.4×10^6^)	123	4.767	1.204–18.883	0.026*
Age (≥65 *vs.* <65)	43	2.620	0.657–10.438	0.172
Logdose	123	1.304	0.579–2.939	0.522

RCC				
Variable	Number of patients^$^	OR	95% CI	p-value(2-sided)
Cellular immune response	75	8.422	1.339–52.966	0.023*
Humoral immune response	12	n.a.		
Median dose (≥38.7 *vs.* <38.7×10^6^)	148	6.973	1.948–24.967	0.003*
Age (≥65 *vs.* <65)	125	1.938	0.718–5.230	0.192
Gender (m *vs.* f)	105	1.134	0.450–2.857	0.790
Logdose	148	4.378	2.265–8.461	0.00*

Abbreviations: OR, odds ratio; CI, confidence interval, n.a., not applicable; m, male; f, female.

§ Analyses restricted to number of patients with available data for the variables listed.

*significant.

**Table 3:** Meta-analysis of influence on clinical benefit rate – chi-square analysis of pooled individual data across studies.

Prostate cancer				
Variable	Number of patients^$^	OR	95% CI	p-value(2-sided)
DC type (mat-moDC *vs.* imm-moDC)**	92	2.800	1.141–6.871	0.023*
DC type (moDC *vs.* density enriched DC)	157	0.202	0.099–0.411	0.000*
DC type (mat-moDC *vs.* density enriched DC)	119	0,300	0.137–0.658	0.002*
Application (id/in/il/sc *vs.* iv)**	84	3.150	1.107–8.963	0.028*

RCC				
Variable	Number of patients^$^	OR	95% CI	p-value(2-sided)
DC type (mat-moDC *vs.* imm-moDC)	154	1.0	0.430–2.324	1.0
Application (id/in/il/sc *vs.* iv)	152	2.188	0.776–6.107	0.132

Abbreviations: OR, odds ratio; CI, confidence interval; mat-moDC, mature monocyte derived dendritic cell; imm-moDC, immature monocyte derived dendritic cell; id, intradermal; in, intranodal; il, intralymphatic; sc, subcutaneous; iv, intraveneous.

§ Analyses restricted to number of patients with available data for the variables listed.

**without density-grade enriched DC.

*significant.

**Figure 3:**
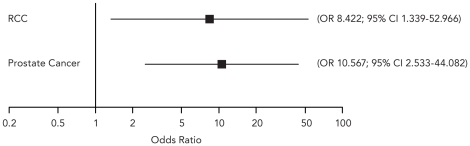
Meta-analysis of the odds ratio (OR) of cellular immune response and clinical benefit rate. Conditional logistic regression grouped by study using individual patient data revealed a statistically significant association between clinical benefit rate and induction of tumor antigen specific cellular immune response for prostate cancer and renal cell cancer (RCC) patients. Horizontal lines denote 95% confidence intervals.

The most relevant finding is that the cellular immune response was found to have a significant influence on CBR, both in *prostate cancer* (OR 10.6, 95% CI 2.5–44.1) and in *RCC* (OR 8.4, 95% CI 1.3–53.0) (Fig. 3).

For assessment of the influence of total DC dose, the median between the trials served as threshold. Strikingly, a significant OR was identified for higher DC doses both in *prostate cancer* and *RCC* trials (*prostate cancer*: OR 4.8, 95% CI 1.2–18.9; *RCC*: OR 7.0, 95% CI 1.9–25.0). To visualize stratified analyses by Forest plots for the influence on clinical outcome of cellular immune response and total DC dose, additional classical Mantel-Haenszel analyses were conducted. Although compared to conditional logistic regression fewer studies and lower patient numbers could be included in these analyses, similar results, which were statistically significant, were obtained (Figure S1).

To further study an association between increasing total DC dose and clinical benefit, the non-dichotomised logarithmic total DC dose (logdose) was assessed by conditional logistic regression. Interestingly, for *prostate cancer* no continuous effect of increasing DC dose could be observed (OR 1.3, 95% CI 0.6–2.9), but there was a statistical significant effect in *RCC* trials (OR 4.4; 95% CI 2.3–8.5).

Specific humoral immune responses were determined only in 12 *RCC* patients. Thus, the analysis was not feasible for *RCC*. In *prostate cancer* patients no statistically significant association of humoral response and CBR was detected (OR 0.8, 95% CI 0.1–4.5). There was no significant association between the variables age in both tumor entities and gender in *RCC* and CBR.

Chi-square tests across the studies revealed a significant positive influence of DC maturation status on the CBR in *prostate cancer* patients (mat-moDC vs. imm-moDC, OR 2.8, 95% CI 1.4–6.9). Interestingly, vaccination routes with access to draining lymph nodes (i.d., i.n., i.l., s.c) were found to result in better clinical response rates in comparison to intravenous (i.v.) injections in both tumor entities. Nevertheless, the OR was statistically significant only in *prostate cancer* patients (OR 3.2, 95% CI 1.1–9.0). Strikingly, comparing monocyte-derived DC with density-enriched DC in *prostate cancer* trials revealed an advantage for density enriched DC over moDC (OR 0.2, 95% CI 0.1–0.4). Even if the comparison was restricted to mat-moDC, the analysis led to the same finding (OR 0.3, 95% CI 0.1–0.7).

To further investigate the influence of DC subtype on CBR, additional conditional logistic regression tests were performed analyzing the variable DC logdose within the subgroups of patients treated with either mature or immature moDC. In *prostate cancer*, for both subgroups, this analysis did not change the statistically not significant result described above. In contrast, in *RCC*, an influence of the increasing DC dose was found only in the group of patients treated with mat-moDC (OR 4.3, 95% CI 2.09–7.79) but not in the group of patients treated with imm-moDC.

## Discussion

A growing body of knowledge about tumor immunosurveillance – and loss thereof – has contributed to the refinement of anti-tumor immunotherapy [64]. Yet, despite high expectations in DC-based vaccination trials, thus far clinical responses have been disappointing [20], [65], [66]. Lack of efficacy can be explained by well-defined tumor escape mechanisms [67]–[69], which are currently being addressed by combining DC vaccination with other approaches, such as CTLA4 [70] or CD25 blockade [52]. However it is also due to the design of clinical trials conducted so far: Most are small phase I/II studies with conventional primary end points safety or feasibility. The objective of this systematic review and meta-analysis was to determine: first, whether an association between immune and clinical response could be detected; and second, which factors were associated with better clinical outcome.

Interestingly, the total objective clinical response rates (7.7% for prostate cancer and 12.7% for RCC) were quite similar compared to DC-based vaccination trials in other tumor entities [20], [65], [71]. When including SD the clinical benefit rate (CBR) was 54% for prostate cancer and 48% for RCC patients, which is consistent with previous reports [14], [16].

Results of meta-analysis of non-randomized phase I/II trials have to be interpreted with caution. For example, the variables dose or DC subtype represent potential confounders to each other. Moreover, definitions of clinical endpoints and immune monitoring are not always consistent among the trials (Tables S1 and S4). Nevertheless, for the key variables (cellular immune response and dose) we could perform conditional logistic regression grouped by study, thus adjusting varying vaccination protocols or response criteria.

Due to the low number of patients with objective responses we had to focus on CBR (CR+PR+MR+SD). However, this clinical endpoint also addresses fundamental aspects of oncology: There is an increasing debate on ideal clinical response criteria in active immunotherapy approaches [72]. Thus far, most trials using DC-vaccines have been conducted in the palliative setting of metastatic or locally advanced disease. For these patients reducing symptoms and prolonging survival time with acceptable side effects are the primary clinical treatment objectives. Thus, overall survival (OS) and time to progression (TTP) would be the ideal endpoints in this situation. This identical challenge has been identified for so called ‘targeted drugs’ [73]. A significant prolongation of median OS was first found in a randomized DC-based trial using density-enriched DC conducted in metastatic prostate cancer [33], which is included in this meta-analysis. It has recently been confirmed by another phase III trial [42] and has led to the FDA approval of Sipuleucel-T in hormone-refractory prostate cancer. When the endpoints OS or TTP are not available, terms of disease stabilization or clinical benefit rate might help evaluation of therapeutic success [20], [74]. However, as shown in our analysis the differences in SD definitions remain problematic (Table S1). Additionally, in nearly one third of the trials included in this study, patients without documented progressive disease at study entry were included. Thus, using CBR as a clinical endpoint will need concise and consistent definitions of SD.

Despite the aforementioned limitations this meta-analysis provides new insights into the field of cancer vaccination. First, we found an association between specific cellular immune response and clinical outcome, both in prostate cancer and RCC. Thus far, this has been reported only within single trials in prostate cancer [22], [37], [43], [46], [48] and RCC [61]. Importantly, this association provides a *proof of concept* for DC-based vaccines. As analyzed in detail, RCC and prostate cancer trials mostly differ in the chosen clinical response criteria and vaccine strategies. For the latter this was particularly observed in the preferred route of vaccination, DC subtype, and different strategies for antigen delivery. Thus, the observed link between induction of cellular immune response and clinical benefit identified by stratified analyses is of great importance due to the fact that is was identified independent of the chosen vaccination strategy and it was, independently of each other, found in both tumor entities.

The second finding relates to the dose of DC. We found a positive influence of higher DC doses on CBR in both tumor entities. The need of a sufficient amount of vaccinated DC to enable activation of effector cells has already been suggested not only in murine models [75], but also in clinical trials [76]. For prostate cancer, Small described an association between DC dose and TTP but failed to show statistical significance [46]. Interestingly, in prostate cancer a positive influence was not found for continuous increasing doses. It seems that a high threshold exists, which has to be exceeded in order to achieve a better clinical response. In RCC also continuous increasing doses had a positive influence on CBR. This difference found between the tumor entities is difficult to interpret, but might be explained by different dose distributions. In addition, it is relevant that in RCC an influence of the increasing DC dose was only found in the group of patients treated with mat-moDC, but not in the group of patients treated with imm-moDC. This finding is of great interest because it further underlines the importance of maturation status as discussed below.

Specific humoral immune response was shown to have no influence on CBR in prostate cancer. However, the number of patients included in this stratified analysis was very low (Table 2). Nevertheless, a chi-square test across the studies that allowed inclusion of a higher number of patients (63) also failed to show a significant association between humoral response and CBR (data not shown).

Our analysis revealed that in prostate cancer patients vaccination of mat- moDC was associated with a higher CBR than vaccination with imm-moDC. For this analysis only monocyte-derived DC were included as density-enriched DC might have a different biology and few information on their maturation status exist. It has been suggested that imm-moDC bear the risk of inducing tolerance [67], [77]–[79]. Indeed, Dhodapkar and colleagues observed tolerizing effects of immature DC to influenza matrix peptide in healthy individuals [80]. In melanoma patients it has been demonstrated that maturation status affects not only lymph-node homing [81], but also the induction of T-cell and thus clinical responses [82], [83]. However, no statistically significant association of maturation status and clinical response has been reported so far. Our finding adds weight to experimental evidence of a tolerizing effect of imm-moDC. This observation could however not be reproduced in RCC – likely due to an inhomogeneous distribution of mat-moDC and imm-moDC within the RCC trials.

Interestingly, comparing the influence on clinical outcome of density-enriched DC with that of monocyte-derived DC in prostate cancer revealed an advantage of density-enriched DC over moDC, even if the comparison was restricted to mat-moDC. This finding might further underscore the FDA approval of the cellular therapeutic Sipuleucel-T [42]. Nevertheless, the limitations of pooled chi-square analyses have to be kept in mind especially for this comparison: using this method there is no adjusting for potential systematic differences between the trials using either DC type. For instance, in trials using density-enriched DC, total DC dose was considerably higher than in trials using monocyte-derived DC. It is only a randomized trial that would allow reliable conclusions about the superiority of either DC type.

The optimal route of vaccination is still subject to debate. DC enter lymph nodes in afferent lymph vessels [84], [85]. Mature DC lack CD62L, a homing receptor necessary to enter lymph nodes across high endothelial venules from the blood [84], [86]. For these reasons a route of vaccination allowing access to draining lymph nodes (i.d., i.l., s.c., i.n) should be superior to an intravenous route. Indeed, murine studies have established that intravenously injected DC accumulate in the spleen and non-lymphoid tissues, but not in the lymph nodes [87]. Our analysis revealed a positive influence on CBR of a non-intravenous route (i.d., i.l., s.c., i.n) compared with an intravenous route for moDC in both cancers, reaching statistical significance only in prostate cancer. This finding confirms observations in single trials that have compared different routes [88]. The statistically not significant result for RCC might be explained by the inhomogeneous distribution of intravenous and non-intravenous routes in RCC trials.

This systematic review indicates that vaccine quality controls clearly need improvement. Notably, only 10 out of 29 trials reported the DC purity, and of these, only in four trials purity was above 80% as propounded by Figdor [62]. Moreover, five clinical trials reported no information about DC phenotype at all. One of the prerequisites for future randomized clinical trials using DC should be a definitive consensus about the vaccine quality controls. Information about toxicity was provided in most of the trials and our analysis further confirms that safety is no general issue in DC-based immunotherapy anymore.

Taken together, our meta-analysis demonstrated an association between specific cellular immune response and clinical benefit, both in prostate cancer and RCC trials, thus implying a proof of concept for DC vaccination. Moreover, this study provides evidence for previous assumptions: Relevance of dose, mature phenotype, and lymph-node access. However, the systematic review also revealed a strong heterogeneity regarding DC purity and dose, DC subtype, antigen delivery, route of vaccination, and quality controls. Therefore, we conclude that DC-based immunotherapy clearly warrants further investigation in phase III randomized trials. It is essential that future trials fulfill quality criteria, e.g. as postulated by Figdor [62] and others [9], [72]. It is important not only to standardize the vaccine itself, but also the immune monitoring and the criteria to assess clinical responses [89]. Only if DC are generated and validated like a drug, can they finally be made available to cancer patients as a standard option.

## Supporting Information

Figure S1 **Forest plots of Mantel-Haenszel odds ratio estimates.** Meta-analysis of the odds ratio of cellular immune response (A, B) or total DC dose (C, D) and clinical benefit rate for prostate cancer and RCC trials are displayed. The size of the squares is proportional to the sample size. Horizontal lines denote 95% confidence intervals for single studies, the diamond the 95% confidence interval for the overall Mantel-Haenszel estimate (fixed effect).
(TIFF)

Table S1 **Additional information about included patients, clinical response criteria and clinical responses.**
(PDF)

Table S2 **Additional information about number of DC per vaccination and number of vaccinations.**
(PDF)

Table S3 **Additional information about toxicity criteria and observed toxicity.**
(PDF)

Table S4 **Detailed information about humoral and cellular immune response testing within the trials.**
(PDF)

Table S5 **Additional information about data extraction for individual patient data level analyses.**
(PDF)

Text S1 **Search string of the Medline Ovid search.**
(PDF)
