# Supplementary figures and images for: Dendritic Cell Based Tumor Vaccination in Prostate and Renal Cell Cancer: A Systematic Review and Meta-Analysis
Source: PLoS One. 2011 Apr 20;6(4):e18801. doi: 10.1371/journal.pone.0018801 (PMC3080391; doi:10.1371/journal.pone.0018801)

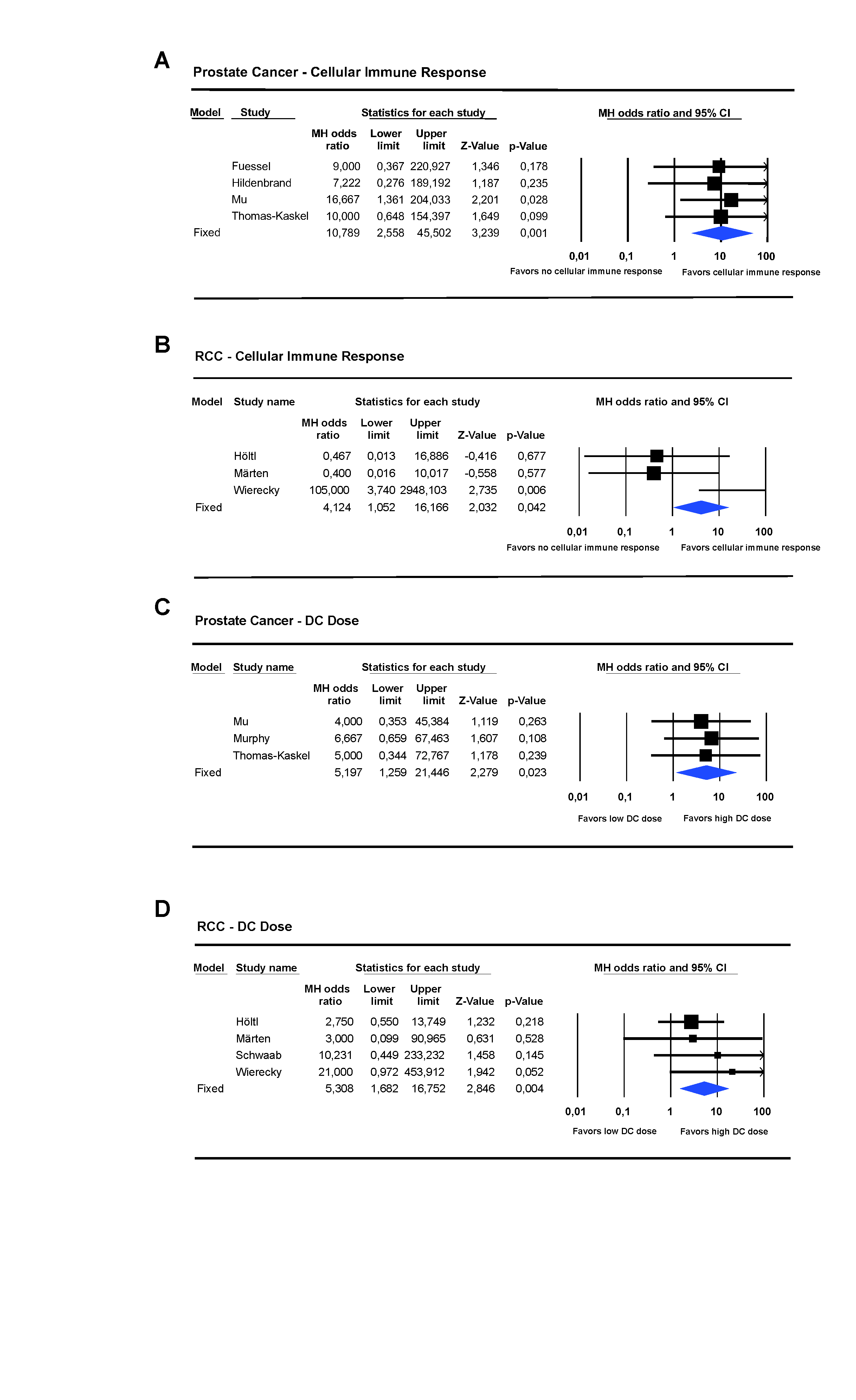

Supplement: Figure S1 — Forest plots of Mantel-Haenszel odds ratio estimates. Meta-analysis of the odds ratio of cellular immune response (A, B) or total DC dose (C, D) and clinical benefit rate for prostate cancer and RCC trials are displayed. The size of the squares is proportional to the sample size. Horizontal lines denote 95% confidence intervals for single studies, the diamond the 95% confidence interval for the overall Mantel-Haenszel estimate (fixed effect). (TIFF) [file pone.0018801.s001.tif]
