# Supplementary material for: Dendritic Cell Based Tumor Vaccination in Prostate and Renal Cell Cancer: A Systematic Review and Meta-Analysis
Source: PLoS One. 2011 Apr 20;6(4):e18801. doi: 10.1371/journal.pone.0018801 (PMC3080391; doi:10.1371/journal.pone.0018801)
Supplement: Table S1 — Additional information about included patients, clinical response criteria and clinical responses. (PDF) [file pone.0018801.s002.pdf]

Table S1 – Additional information about clinical responses

Reference	Patients	Progressive disease before study enrollment?	Clinical response criteria	Information about response evaluation	CR	PR	MR	SD	PD	Additional information about clinical response	Additional information about clinical response interpretation
**Prostate**											
Barrou, 2004	26 BCR<br>(24 vaccinated; 1 patient too low DC number, 1 patient refused)	mandatory	PSA course					11	13	Molecular tumor cell clearence in 6/6 investigated patients (3 of these with PD)	Stable diseases concluded analogue PSA-WG criteria (PSA drop <40%)
Burch, 2000	13 metastatic	mandatory	NPCP/PSA-WG analogue	Restaging 4 weeks after last vaccination, then every 8 weeks		3		9	1	Radiologically no change	PR: patients with PSA drop >50%<br>SD: concluded from TTP (TTP 30–274 days after registration)
Fong, 2001	21 metastatic	mandatory	NPCP/PSA-WG analogue	Restaging 4 months after vaccination				6	15	6 x PSA drop<br>SD confirmed by CT and bone scans<br>Correlation between clinical responses and T-cell responses	
Fuessel, 2006	7 metastatic +<br>1 BCR	mandatory	NPCP/PSA-WG analogue	Restaging 2 weeks and 3 months after last vaccination		1		3	4	Radiologically no change	
Heiser, 2002	16 metastatic<br>(13 evaluable)	not mandatory	PSA log slope							1/7 PSA decrease + 5/7 PSA log slope reduction<br>3 patients with transient molecular clearence	
Higano, 2009	65 metastatic +<br>33 in placebo group (APC without PA2024)	mandatory	TTP analogue WHO criteria	Restaging every 8 weeks until week 32, then every 12 weeks						TTP treatment vs. placebo group: 10.9 vs. 9.9 months (ns)<br>OS: 19.0 vs. 15.7 months (ns) (after adjusting baseline imbalances of LDH, PSA, localistion of disease, number of bone metastases, and weight OS improvement was significant)	
Hildenbrand, 2007	12 metastatic	mandatory	NPCP/PSA-WG analogue	Restaging 3 weeks after last vaccination		1	1	4	6	PR and MR due to radiological response<br>PSA course: PSA decrease in 2 patients (1 PR, 1 SD) , PSA velocity decrease in 4 patients, PSA velocity stable in 1 patient, PSA velocity increase in 5 patients	
Kantoff, 2010	341 metastatic +<br>171 in placebo grpup (APC without PA2024)	mandatory	OS and TTP analogue WHO criteria	Restaging 2, 10, 22 and 30 weeks after last vaccination, thereafter every12 weeks						OS treatment vs. placebo group: 25.8 vs. 21.7 months (sig)<br>TTP: 14.6 vs. 14.4 months (ns)<br>OSR (36 months): 31.7% vs. 23.0%<br>1 PR in treatment group<br>PSA course: reduction > 50% in 8/311 treated patients vs. 2/153 in placebo group	
Mu, 2005	14 metastatic +<br>6 BCR (5 vaccinated, 1 patient progressive disease before vaccination)	mandatory	NPCP/PSA-WG analogue	First assessment 2 weeks after last vaccination (bone scan: 9 weeks after last vaccination)				11	8	PSA course: decreased PSA log slope in 13 patients<br>T-cell responses correlate with clinical response	
Murphy, 2000	28 metastatic<br>(of these, 11 patients previously vaccinated with lower DC dose; mean time since previous last vaccination 405 days)	not mandatory	NPCP/PSA-WG analogue	No information about time of restaging	1	4		1 (previous PR)	21		1 patient with previous CR before re-vaccination was excluded from the systematic review (the authors judged it as CR)
Pandha, 2004	9 metastatic<br>2 BCR	not mandatory	RECIST criteria<br>(for PD also PSA course used)	First evaluation 4 weeks after last vaccination				4	7	PSA course:<br>PSA decrease in 1/11 patients, PSA stable in 1/11 patients, PSA velocity reduction in 1/11 patients<br>increased PSA doubling time in 6/11 patients	
Perambakam, 2006	7 metastatic<br>7 locally advanced	not mandatory	n.d.	n.d.						n.d.	n.d.
Small, 2000	12 metastatic<br>19 BCR	mandatory	PSA course, additionally radiographic courses described	PSA course every 4 weeks		3		28		Patients with PR radiologically stable<br>Correlation between cellular immune response and TTP and between number of DC and TTP	SD: concluded from TTP (always ≥ 8 weeks)
Small, 2006	82 metastatic +<br>45 in placebo group (APC without PA2024)	mandatory	TTP analogue WHO criteria	Restaging every 8 weeks until week 32, then every 12 weeks						TTP treatment group vs. placebo group: 11.7 vs. 10.0 months (ns)<br>OS: 25.9 vs. 21.4 months (sig)<br>OSR (36 months): 34% vs. 11% (sig)<br>[previous publication (Lee, 2003): TTP significant better for patients with Gleason score < 7]	
Su, 2005	20 metastatic	not mandatory	PSA course, but only median information for vaccination groups	PSA course biweekly for 6 weeks, then after one month, afterwards every 4 months						PSAdt improved in group with higher number of vaccinations<br>Molecular micrometastases clearing (RNA) in 4/4 evaluable patients of LAMP-hTERT and 5/6 patients of hTERT group<br>"no objective clinical response"	
Thomas-Kaskel, 2006	12 metastatic	mandatory	NPCP/PSA-WG analogue	Restaging 2 weeks after last vaccination				6	6	PSA course: 4/12 patients with significant decrease in log PSA slope;<br>1/12 patient with PSA decrease (all SD)<br>OS correlated with positive DTH	
Waeckerle-Men, 2006	6 metastatic	mandatory	PSA course, additionally radiographic courses described	Restaging after last vaccination, 6 weeks thereafter, and then every 3 months					3	Clinical course (PD) specified only for 3 patients<br>PSA-doubling time improved in 3 immunological responding patients;<br>1 patient with transient PSA decrease	

Table S1 – Additional information about clinical responses

Reference	Patients	Progressive disease before study enrollment?	Clinical response criteria	Information about response evaluation	CR	PR	MR	SD	PD	Additional information about clinical response	Additional information about clinical response interpretation
**RCC**											
Berntsen, 2008	30 metastatic (27 evaluable: 2 patients died before first vaccination, 1 patient withdrew informed consent after 4th vaccination)	mandatory	RECIST criteria	Restaging after 6th and 10th vaccination and then every 3 months				13	14	According to RECIST SD categorization only if duration was ≥ 8 weeks<br>duration of SD: 10/13 patients >6 mo, 5/13 patients > 12mo<br>median OS: 16.6 mo; median PFS: 2.7 mo	
Bleumer, 2007	8 metastatic (6 evaluable)	mandatory	WHO/RECIST criteria suggested	Restaging 4 weeks after last vaccination					6		
Dannull, 2005	10 metastatic	not mandatory	n.s.	no information on clinical response						follow-up interval from last vaccination to last staging follow-up specified and classification of patients to be alive with disease (AWD) or dead of disease (DOD)	
Gitlitz, 2003	14 metastatic (12 vaccinated + 2 patients progressive before vaccination)	enrollment before nephrectomy	WHO analogue	Restaging 3 months after last vaccination and every 3 months		1		3	8		
Höltl, 2002	35 metastatic	enrollment before nephrectomy	WHO/RECIST criteria suggested (described responses in accordance with WHO criteria)	Restaging 1 month after each vaccination	2	1		7	25	8 PD before 3rd vaccination	
Kim, 2007	9 metastatic	enrollment before nephrectomy	WHO criteria	Restaging 4 weeks after each vaccination cycle		1		5	3		
Märten, 2002	15 metastatic (only 11 patients vaccinated with tumor lysate pulsed DC)	mandatory	WHO criteria	Restaging 3 weeks after last vaccination		1		7	7	2 patients with SD received only KLH pulsed DC and 2 patients with PD received unpulsed DC; 2 patients with tumor lysate pulsed DC (1 SD, 1 PD) and 1 patient with KLH only pulsed DC received additionally IFN, IL-2 and 5-FU after the end of vaccination period	For individual analysis only patients with tumor lysate pulsed DC included
Oosterwijk-Wakka, 2002	12 metastatic	enrollment before nephrectomy	WHO/RECIST criteria suggested	Restaging 3 weeks after last vaccination, follow-up at 6 weeks intervals				8	4		
Schwab, 2009	18 metastatic	mandatory	RECIST criteria	Follow-up for responding and stable patients every 3 months	3	6		6	3	responses occured after induction (first 2 vaccines) or after maintenance vaccination (vaccination 3-5); information on 'best response' (without specification of time point)	
Su, 2003	15 metastatic (10 evaluable)	not mandatory	Not specified (clinical course described)					1 (> 22mo)	1	Remaining 8 patients received further therapy	
Wei, 2007	10 metastatic	not mandatory	WHO/RECIST suggested (described resonses in accordance with WHO criteria)	Restaging 3 months after last vaccination		1		3	6		
Wierecky, 2006	20 metastatic	17/20 patients progressive, 2/20 not specified, 1/20 stable disease	WHO criteria	Restaging between 1 and 2.5 weeks after last vaccination and every month thereafter	1	2	2	5	10	Stronger cellular immune response in clinical responding patients	
