# Supplementary material for: Dendritic Cell Based Tumor Vaccination in Prostate and Renal Cell Cancer: A Systematic Review and Meta-Analysis
Source: PLoS One. 2011 Apr 20;6(4):e18801. doi: 10.1371/journal.pone.0018801 (PMC3080391; doi:10.1371/journal.pone.0018801)
Supplement: Table S2 — Additional information about number of DC per vaccination and number of vaccinations. (PDF) [file pone.0018801.s003.pdf]

Table S2 – Additional information about dose

Prostate		
**Reference**	**Number of DC per vaccination**	**Mean number of vaccinations (range)**
Barrou, 2004	14.6 – 72.2 × 10^6	9
Burch, 2000	mean 255 × $10^6/m^2$	2 (1 – 2)
Fong, 2001	mean 11.2 × 10^6	2
Fuessel, 2006	10 × 10^6 i.d. + 10 × 10^6 i.v.	4
Heiser, 2002	3 patients 10 × 10^6 i.v. + 10 × 10^6 i.d.<br>4 patients 30 × 10^6 i.v. + 10 × 10^6 i.d.<br>6 patients 50 × 10^6 i.v. + 10 × 10^6 i.d.	3
Higano, 2009	no information	3
Hildenbrand, 2007	6 × 10^6 (2 × 10^6 for each antigen)	4
Kantoff, 2010	'minimum' 40 x 10^6	3
Mu, 2005	20 × 10^6	4 (4 – 6)
Murphy, 2000	mean 28.8 × 10^6 (21.0 – 29.3 × 10^6)	3 (1 – 4)
Pandha, 2004	1 – 3 × 10^6	6
Perambakam, 2006	median 155.5 × 10^6<br>mean 149.9 × 10^6	3
Small, 2000	median 123 × 10^6 (1.4 – 1276 × 10^6)	3 (3 – 4)
Small, 2006	median 3650 × 10^6 cells (745 × 10^6 $CD54_{high}$ cells)	3
Su, 2005	10 × 10^6	3 (3 – 6)<br>(1 group 3 vaccinations; 1 group 6 vaccinations)
Thomas-Kaskel, 2006	median 27 × 10^6 (8 – 154 × 10^6)<br>mean 37.8 × 10^6	4 (3 – 7)
Waeckerle-Men, 2006	5 × 10^6	9 (3 – 18)<br>(6 × biweekly + monthly booster until PD)

RCC		
**Reference**	**Number of DC per vaccination**	**Mean number of vaccinations (range)**
Berntsen, 2008	10 x 10^6	6 (4 - 29)
Bleumer, 2007	mean 56 × 10^6	5
Danull, 2005	10 x 10^6	3
Gitlitz, 2003	mean 4.59 × 10^6	4 (3 – 4)
Höltl, 2002	mean 8.1 × 10^6 (1.3 – 38 × 10^6)	3 (3 – 13)
Kim, 2007	3 patients 10 × 10^6<br>6 patients 50 × 10^6	8 (4 – 8)<br>Responding patients received 4 further vaccinations
Märten, 2002	median 3.76 × 10^6<br>mean 4.77 × 10^6	5
Oosterwijk-Wakka, 2002	mean 12.9 × 10^6	3
Schwaab, 2009	10 x 10^6	3.5 (1 - 5)
Su, 2003	8 patients 10 × 10^6 i.v. + 10 × 10^6 i.d.<br>2 patients 30 × 10^6 i.v. + 10 × 10^6 i.d.	3
Wei, 2007	median 0.58 × 10^6<br>mean 0.65 × 10^6	3 (1 – 6)
Wierecky, 2006	median 2.69 × 10^6<br>mean 3.62 × 10^6	8 (2 – 28)
