# Supplementary material for: Dendritic Cell Based Tumor Vaccination in Prostate and Renal Cell Cancer: A Systematic Review and Meta-Analysis
Source: PLoS One. 2011 Apr 20;6(4):e18801. doi: 10.1371/journal.pone.0018801 (PMC3080391; doi:10.1371/journal.pone.0018801)
Supplement: Table S3 — Additional information about toxicity criteria and observed toxicity. (PDF) [file pone.0018801.s004.pdf]

Table S3 – Additional information about toxicity

Reference	Toxicity criteria	Toxicity
**Prostate**		
Barrou, 2004	NIH/NCI-CTC	4 patients grade 1/2 (rash, asthenia, halitosis)
Burch, 2000	NIH/NCI-CTC	5 patients fever grade 1/2<br>5 patients local reactions grade 1/2<br>5 patients myalgia grade 1/2
Fong, 2001	NIH/NCI-CTC	2 patients fever grade 1/2<br>3 patients local reactions grade 1/2<br>1 patient painful swollen LN grade 1/2<br>5 patients with elevated ANA-titer and 1 patient with elevated RF ('clinical irrelevant')
Fuessel, 2006	No grading	No immediate or delayed toxicity
Heiser, 2002	Not specified but toxicities graded	4 patients fever grade 1/2 (with flu-like symptoms)<br>4 patients local reactions grade 1/2
Higano, 2009	NIH/NCI-CTC	Only information on integrated data including trial D9901 (Small, 2006): Events occuring at higher rate than in placebo group were chills, pyrexia, headache, ashenia, dyspnea, vomiting, and tremor (primarily grade 1/2). Any adverse event grade 3/4 33.3% in treatment vs. 27.6% in placebo group (ns).<br>'Possibly increased risk' of cerebrovascular events in treatment group (7.5%) compared to 2.6% in placebo group.
Hildenbrand, 2007	NIH/NCI-CTC	11 patients fever grade grade 1/2<br>11 patients local reactions grade 1/2<br>1 patient insomnia grade 3<br>6 patients bone pain grade 1/2<br>3 patients articular pain grade 1/2
Kantoff, 2010	NIH/NCI-CTC	Events reported more frequently in treatment than in placebo group were chills, fever, headache, inluenza-like illness, myalgia, hypertension, hyperhydrosis, groin pain<br>Adverse events grade 3 or higher in 23/338 patients of treatment group vs. 3/168 patients of placebo group.<br>No difference for cerebrovascular events in treatment group (8/337 patients) vs. placebo group (3/168 patients).
Mu, 2005	NIH/NCI-CTC	'Some local reactions' grade 1
Murphy, 2000	Not specified	'Minimal' (fever, fatigue, muscle cramps)
Pandha, 2004	Not specified	'Minimal local reactions'
Perambakam, 2006	n.e.	n.e.
Small, 2000	NIH/NCI-CTC	13 patients fever grade 1/2<br>2 patients fever grade 3/4<br>2 patients myalgia grade 1/2<br>5 patients urinary complains grade 1/2
Small, 2006	NIH/NCI-CTC	49 patients rigor, 32 fatigue, 24 pyrexia, 8 tremor, 7 feeling cold<br>20 patients with toxicity grade 3/4<br>'More toxicities in treated group than in placebo group'
Su, 2005	Analogue NIH/NCI-CTC	18 patients local reactions grade 1/2<br>4 patients fatigue or flu-like symptoms grade 1/2
Thomas-Kaskel, 2006	WHO criteria	No immediate or delayed toxicity
Waeckerle-Men, 2006	Not specified, but toxicities graded	1 patient fever grade 1/2<br>5 patients local reactions grade 1/2<br>1 patient nausea grade 1/2

RCC		
**Reference**	**Toxicity criteria**	**Toxicity**
Berntsen, 2008	NIH/NCI-CTC	17 patients infuenza-like illness grade 1/2<br>16 patients fatigue grade 1/2<br>9 patients fever grade 1/2<br>9 patients local reactions grade 1/2<br>7 patients nausea grade 1/2<br>5 patients sweating grade 1/2<br>5 patients coughing grade 1/2<br>3 patients vomiting grade 1/2<br>2 patients rash grade 1/2<br>1 patient depression grade 1/2
Bleumer, 2007	Not specified, but toxicities graded	Some flu-like symptoms<br>Local reactions in 'most' patients, never > grade 2
Danull, 2005	Not specified	'Without any major clinical toxicities or evidence of autoimmunity'.
Gitlitz, 2003	NIH/NCI-CTC	3 patients fatigue grade 1/2<br>1 patient nausea grade 1/2<br>1 patient diarrhea grade 1/2
Höltl, 2002	Not specified but toxicities graded	8 patients fever grade 1/2
Kim, 2007	NIH/NCI-CTC	7 patients fever grade 1/2<br>9 patients local reactions grade 1/2<br>9 patients with myalgia, headache, skin rash, lipase elevation or renal insufficiency grade 1/2
Märten, 2002	Not specified	No toxicity
Oosterwijk-Wakka, 2002	NIH/NCI-CTC	5 patients local reactions grade 1/2
Schwaab, 2009	Not specified, but toxicities graded	Toxicities interpreted as IL2 and IFN-α2a related; grade 3/4:<br>9 patients rash with or without pruritus<br>6 patients hypotension or hypertension<br>10 patients electrolyte or metabolic changes<br>3 patients cytopenia<br>1 patient myalgia<br>3 patients confusion<br>3 patients infection<br>4 patients edema<br>1 patients pulmonary toxicity<br>2 patients renal function<br>3 patients cardial side effects<br>1 patient deep veneous thrombosis<br>1 patient diarrhoe<br>1 patient nausea<br>1 patient syncope<br>In two patients toxicity interpreted as autoimmune syndrome (therefore 1 patient removed before his 5th cycle).
Su, 2003	Not specified but toxicities graded	5 patients local reactions grade 1/2
Wei, 2007	Adverse events (AE) described	9 patients with fever<br>Variety of other toxicities 'most of these disease or IL-2 related'<br>No SAE
Wierecky, 2006	Not specified	Not described
