# Supplementary material for: Dendritic Cell Based Tumor Vaccination in Prostate and Renal Cell Cancer: A Systematic Review and Meta-Analysis
Source: PLoS One. 2011 Apr 20;6(4):e18801. doi: 10.1371/journal.pone.0018801 (PMC3080391; doi:10.1371/journal.pone.0018801)
Supplement: Table S4 — Detailed information about humoral and cellular immune response testing within the trials. (PDF) [file pone.0018801.s005.pdf]

Table S4 – Immune Responses

Prostate				
Reference	Humoral immune response		Cellular immune response	
	Type of response	n/total investigated	Type of response	n/total investigated
Barrou, 2004	ELISA (PSA Ab)	0/24	ELISPOT (rPSA)<br>ELISPOT (PSA peptides)	2/24 (3/22 before vaccination)<br>9/14 (5/12 before vaccination)
Burch, 2000	ELISA (PA2024)<br>ELISA (PAP)<br>ELISA (GM-CSF)	11/11<br>5/11<br>10/11	Proliferation (PA2024)<br>Proliferation (PAP)<br>Proliferation (GM-CSF)	9/9<br>9/9<br>9/9
Fong, 2001	ELISA (mPAP)<br>ELISA (hu PAP)	8/21<br>6/21	IFN-γ supernatant T-cell stimulation (mPAP)<br>ELISPOT (huPAP)<br>Proliferation (mPAP)<br>Proliferation (huPAP)	7/18<br>8/18<br>21/21<br>11/21
Fuessel, 2006	n.d.		ELISPOT (PSMA + monocytes)<br>ELISPOT (prostein + monocytes)<br>ELISPOT (survivin + monocytes)<br>ELISPOT (PSA or Trp-8 + monocytes)	2/8<br>3/8<br>2/8<br>0/8
Heiser, 2002	ELISA (PSA)	0/13<br>(but ‘data not shown’)	ELISPOT (PSA tDC as stimulators)<br>Cytotoxicity (PSA tDC as stimulators)	8/8<br>10/10
Higano, 2009	n.d.		n.d.	
Hildenbrand, 2007	n.d.		DTH (DC-PSA-1-3); < 5 mm ('negative')<br>DTH (DC-PSA-1-3); 5 – <10 mm (‘weak positive’)<br>DTH (DC-PSA-1-3); 10 – <12 mm ('positive')<br>DTH (DC-PSA-1-3); ≥ 12 mm ('strong positive’)	2/12<br>1/12<br>4/12<br>5/12
Kantoff, 2010	ELISA (PA2024)<br>ELISA (PSA)	100/151 (2/70 in placebo group)<br>43/151 (1/70 in placebo group)<br><br>(sig. higher OS in patients with PA2024- or PSA-antibody titer >400)	T-cell proliferation (PA2024)<br>T-cell proliferation (PSA)	46/63 (4/33 in placebo group)<br>15/55 (2/25 in placebo group)<br><br>(no OS difference in patients with or without PA2024/PSA T-cell proliferation responses)
Mu, 2005	n.d.		ELISPOT (T-cell enriched PBMC + tumor RNA tDC)<br>Proliferation (T-cell enriched PBMC + tumor RNA tDC)<br>DTH (tumor RNA)<br>Cytotoxicity (tumor RNA tDC and PC-3 cell line)	10/19<br>9/19<br>5/16<br>1/1
Murphy, 2000	n.d.		n.d.	
Pandha, 2004	ELISA (KLH)	10/11	DTH (lysate)<br>DTH (KLH)<br>DTH biopsy (lymphocyte infiltration; lysate)<br>DTH biopsy (lymphocyte infiltration; KLH)<br>ELISPOT (lysate)<br>ELISPOT (KLH)<br>Proliferation (lysate)<br>Proliferation (KLH)<br>IFN-γ supernatant DC/lysate pulsed PBMC<br>IL-10 supernatant DC/lysate pulsed PBMC<br>IFN-γ supernatant KLH pulsed PBMC<br>IL-10 supernatant DC/lysate pulsed PBMC	0/11<br>1/11<br>10/11<br>10/11<br>6/11<br>not specified (“less”)<br>10/11<br>10/11<br>4/8<br>4/8<br>1/8<br>4/8
Perambakam, 2006	n.d.		DTH (PSA)<br>DTH (Flu-M1)<br>Cytokine bead assay (PSA stimulation of DTH infiltrating T cells):<br>- IFN-γ<br>- TNF-α	5/14<br>12/14<br><br><br>2/2<br>1/2
Small, 2000	ELISA (PAP)<br>ELISA (GM-CSF)	16/31<br>25 /31<br>(10 preexisting)	Proliferation (PA2024)<br>Proliferation (KLH)<br>Proliferation (PAP)<br>Proliferation (GM-CSF)<br>ELISPOT (PA2024)	31/31<br>5/5<br>10/26<br>19/27 (15 preexisting)<br>2/2
Small, 2006	n.d.		Proliferation (PA2024)	31 patients investigated in ‘treated group’, 18 patients investigated in ‘placebo group’: T-cell proliferation index 8 times higher in ‘treated group’

Prostate	(cont'd)			
Reference	Humoral immune response		Cellular immune response	
	Type of response	n/total investigated	Type of response	n/total investigated
Su, 2005	n.d.		ELISPOT (hTERT or LAMPhTERT; either $CD4^+$ or $CD8^+$)	17/18
			DTH (hTERT)	9/11
			DTH (LAMPhTERT)	9/9
			Cytotoxicity (hTERT; T cells from DTH biopsies)	1/1
			Cytotoxicity (hTERT; PBMC)	4/4 (LAMP group stronger)
			PSA-RNA clearance in PB	9/10
Thomas-Kaskel, 2006	n.d.		Tetramer (PSCA)	1/11
			Tetramer (PSA)	1/11
			Tetramer (HIVgag)	1/11
			DTH (PSCA)	5/10
			DTH (PSA)	3/10
			DTH (HIVgag)	1/10
Waeckerle-Men, 2006	n.d.		ELISPOT (PSCA)	3/4
			ELISPOT (PAP, PSMA, PSA)	3/4
			ELISPOT (FluM/TT)	3/4
			Tetramer (PSCA)	3/4
			Tetramer (FluM)	3/4
			DTH (PSCA /PSA/PAP/PSMA)	0/3
			DTH (FluM/TT)	0/3
			Cytotoxicity (PSCA)	3/3
			Cytotoxicity (FluM)	3/3

RCC				
Reference	Humoral immune response		Cellular immune response	
	Type of response	n/total investigated	Type of response	n/total investigated
Berntsen, 2008	n.d.		ELISPOT (survivin, telomerase)<br>DTH (antigen pulsed DC)<br>Tetramer (hTERT) in 1 patient (prolonged SD)	6/6<br>6/19<br>1/1
Bleumer, 2007	ELISA (KLH)<br><br>ELISA (CA9)	6/6<br><br>0/6	IL-5/IL-13 release in supernatant of T-cell stimulation assay (DC + KLH + CA9 peptides)<br>DTH (DC+KLH)<br>DTH (DC+KLH+CA9p249)<br>DTH (DC+KLH+CA9p254)	4/6<br><br>6/6<br>6/6<br>5/6
Dannul, 2004	n.d.		ELISPOT (enriched CD4 and CD8 T cells + tumor RNA transfected DC)	9/10 (failed in 1 patient without T_{Reg} depletion; stronger in T_{Reg} depleted patients compared to patients without T_{Reg} depletion, significant for $CD8^+$ Tcells)
Gitlitz, 2003	n.d.		Cytotoxicity (autologous tumor)<br>Cytotoxicity (cell line)	0/12<br>0/12
Höltl, 2002	n.d.		Proliferation (KLH)<br>DTH (KLH)<br>Proliferation (OFA/LRP)<br>IFN-γ release of OFA/LRP stimulated PBMC<br>IL-10 release of OFA/LRP stimulated PBMC	11/11<br>4/4<br>5/6<br>3/6<br>1/6
Kim, 2007	n.d.		DTH (tumor lysate + DC)<br>DTH (KLH + DC)<br>Proliferation (PBL + tumor lysate + KLH)<br>ELISPOT (PBL + KLH)	6/9<br>6/9<br>8/9<br>1/2
Märten, 2002	n.d.		DTH (tumor lysate)<br>DTH (KLH)<br>Additional ELISPOT-, proliferation- and cytotoxicity-assays have been reported, but without specifying stimulation	2/9<br>2/3
Oosterwijk-Wakka, 2002	ELISA (tu lysate)<br>ELISA (KLH)	0/12<br>0/6	Proliferation (tumor lysate)<br>DTH (tumor lysate)<br>Proliferation (KLH)<br>ELISA supernatant KLH stimulated PBMC	0/12<br>0/12<br>6/6 (“low”)<br>6/6 (“low” and “data not shown")
Schwaab, 2009	indirect detection of anti-RCC antibodies via secondary anti-human antibodies after binding to RCC cell lines	"significant increase of IgM but not IgG antibodies" (pooled analysis across investigated patients)	Proliferation (PBL + tumor lysate loaded DC)	"increase of RCC specific $CD8^+IFN\gamma^+$ T cells", not significant (pooled analysis across investigated patients)
Su, 2003	n.d.		ELISPOT (RCC RNA)<br>Cytotoxicity<br>ELISPOT (OFA, hTERT, and G250)	6/7<br>1/1<br>“weak responses”
Wei, 2007	n.d.		IFN-γ intracellular staining (PBMC + autologous tumor lysate) of $CD4^+$ and $CD69^+$T cells<br>IFN-γ intracellular staining (PBMC + autologous tumor lysate) of $CD8^+$ and $CD69^+$T cells<br>IFN-γ supernatant PBMC + autologous tumor lysate	9/9<br><br>5/8<br><br>“significant at 2 weeks after 1st vaccination” (pooled analysis across investigated patients)
Wierecky, 2006	n.d.		ELISPOT (MUC-1)<br>Proliferation (PADRE)<br>Cytotoxicity (MUC-1)<br>ELISPOT (survivin, telomerase, G250, or OFA)	4/4<br>10/18<br>11/18<br>6/8
