# Supplementary material for: Dendritic Cell Based Tumor Vaccination in Prostate and Renal Cell Cancer: A Systematic Review and Meta-Analysis
Source: PLoS One. 2011 Apr 20;6(4):e18801. doi: 10.1371/journal.pone.0018801 (PMC3080391; doi:10.1371/journal.pone.0018801)
Supplement: Table S5 — Additional information about data extraction for individual patient data level analyses. (PDF) [file pone.0018801.s006.pdf]

Table S5 – Additional information on individual data extraction

Prostate				
**Reference**	**Patients individually analysed**	**Clinical response**	**Number of DC and number of vaccinations / dose**	**Immune response**
**Barrou, 2004**	24	No individual analysis	Fixed number of vaccinations<br>Median number of DC calculated	Individual information in table
**Burch, 2000**	13	Information in results section and figures	Mean dose of treatment groups	Individual information in results section and figures
**Fong, 2001**	21	Individual information in table	Individual information in table	Individual information in table
**Fuessel, 2006**	8	Individual information in table	Fixed dose	Individual information in table
**Heiser, 2002**	13	No individual analysis	Fixed dose	Individual information in table and figures
**Higano, 2009**		No individual analysis	No individual analysis	No individual analysis
**Hildenbrand, 2007**	12	Individual information in table	Fixed dose	Individual information in table
**Kantoff, 2010**		No individual analysis	No individual analysis	No individual analysis
**Mu, 2005**	19	Individual information in table	Fixed dose	Individual information in table
**Murphy, 2000**	27	Individual information in table only for responders (other patients 'all PD')	Individual information in table only for responders, for other patients mean numbers of vaccinated DC and exact numbers of vaccinations	Not investigated
**Pandha, 2004**	11	Individual information in table	Median number of DC<br>Fixed number of vaccinations	Individual information in results section, tables and figures
**Perambakam, 2006**	14	Not investigated	Median number of DC<br>Fixed number of vaccinations	Individual information in table
**Small, 2000**	31	Results section and conclusion from TTP for SD	Median number of DC and vaccinations	Information in results section
**Small, 2006**		No individual analysis	No individual analysis	No individual analysis
**Su, 2005**	20	No individual information	Fixed dose	Individual information in table
**Thomas-Kaskel, 2006**	12	Individual information in table	Individual information in table	Individual information in table
**Waeckerle-Men, 2006**	6	Individual information in results section, but PD specified only for 3 patients	Fixed dose	Individual information in results section and figures

RCC				
**Reference**	**Patients individually analysed**	**Clinical response**	**Number of DC and number of vaccinations / dose**	**Immune response**
**Berntsen, 2008**	27	Individual information in table	Individual information in table, fixed dose per vaccination	No individual analysis
**Bleumer, 2007**	6	Infomation in results section	Mean number of DC<br>Fixed number of vaccinations	Individual information in table
**Dannull, 2005**	10	No individual analysis	Fixed dose	Individual information in results section, table and figures
**Gitlitz, 2003**	12	Individual information in table	Individual information in table	Information in results section
**Höltl, 2002**	27	Individual information in table	Individual information in table	Individual information in results section and figures
**Kim, 2007**	9	Individual information in table	Fixed dose	Individual information in table
**Märten, 2002**	11	Individual information in table; only patients with tumor lysate pulsed DC included	Individual information in table	Individual information in table
**Oosterwijk-Wakka, 2002**	12	Individual information in table	Fixed number of vaccinations Mean number of DC	Information in results section
**Schwaab, 2009**	18	Individual information in table	Individual information in table, fixed dose per vacciantion	No individual analysis
**Su, 2003**	10	Individual information for 2 patients (other patients received additional treatment)	Fixed dose	Individual information in table
**Wei, 2007**	10	Individual information in table	Individual information in table	Individual information in results section and figures
**Wierecky, 2006**	20	Individual information in table	Individual information in table	Individual information in table
