# Supplementary material for: Dendritic Cell Based Tumor Vaccination in Prostate and Renal Cell Cancer: A Systematic Review and Meta-Analysis
Source: PLoS One. 2011 Apr 20;6(4):e18801. doi: 10.1371/journal.pone.0018801 (PMC3080391; doi:10.1371/journal.pone.0018801)
Supplement: Text S1 — Search string of the Medline Ovid search. (PDF) [file pone.0018801.s007.pdf]

Text S1 - Search String Medline-Ovid

1. (dendritic adj2 cell$).tw,kf,ot
2. Dendritic-cell$.tw,kf,ot
3. (cell-based adj2(immuno?therap$ or vaccinat$)).tw,kf,ot
4. Or/1-3
5. Exp NEOPLASM BY HISTOLOGIC TYPE/
6. Exp NEOPLASM BY SITE/
7. neoplas$.tw,kf,ot.
8. tumo?r$.tw,kf,ot.
9. cancer$.tw,kf,ot.
10. metasta$.tw,kf,ot.
11. malignan$.tw,kf,ot.
12. carcino$.tw,kf,ot.
13. sarcom$.tw,kf,ot.
14. leuk?#m$.tw,kf,ot.
15. lymphom$.tw,kf,ot.
16. melanom$.tw,kf,ot.
17. (gliom$ or glioblastom$).tw,kf,ot.
18. osteo?sarcom$.tw,kf,ot.
19. neuroblastom$.tw,kf,ot.
20.(mesothelio$ or mesotelio$).tw,kf,ot.
21. or/5-2022. Exp CLINICAL TRIALS/
23. (phase I or phase II or phase III).tw,kf,ot.
24. Controlled clinical tral.pt.
25. Clinical trail.pt.
26.(clin$ adj10 trial$).ti,ab.
27. (pilot adj5 stud$).ti,ab.
28. (feasibility adj5 stud$)ti,ab.
29. (monitoring adj5 (stud$ or trial$)).ti,ab.
30. research design/
31. exp evaluation studies/
32. follow up studies/
33. Prospective studies/
34. (control$ or prospectiv$).ti,ab.
35. or/22-34
